# The relationship between family cohesion and bedtime procrastination among Chinese college students: the chain mediating effect of coping styles and mobile phone addiction

**DOI:** 10.1186/s12888-024-05700-8

**Published:** 2024-04-02

**Authors:** Jiahao Huang, Zhenliang Yang, Qian Wang, Junling Liu, Wenlan Xie, Yuqi Sun

**Affiliations:** 1 School of Mental Health, Zhejiang Provincial Clinical Research Center for Mental Disorders, The Affiliated Wenzhou Kangning Hospital, Wenzhou Medical University, 325035 Wenzhou, China; 2 Zhejiang Haozhonghao Health Products Co., Ltd, 325409 Wenzhou, China; 3 Zhejiang Jerinte Health Technology Co., Ltd, 310013 Hangzhou, China; 4 Faculty of Psychology, Tianjin Normal University, 300387 Tianjin, China; 5 Children’s Research Institute, Ningbo Childhood Education College, 315000 Ningbo, China

**Keywords:** Bedtime procrastination, Family cohesion, Coping style, Mobile phone addiction, Chinese college students

## Abstract

**Background:**

Bedtime procrastination refers to an individual’s inability to go to bed at a predetermined time without external obstacles. Previous researchers have found that the bedtime procrastination is harmful to human physical and mental health, but these research on bedtime procrastination have mostly focused on exploring individual factors, while ignoring the external environmental factors. Therefore, this is the first study to investigate bedtime procrastination from the perspective of family environments.

**Methods:**

The study was conducted using a convenient sampling method and online questionnaires. Family Cohesion Scale, Coping Styles Questionnaire, Mobile Phone Addiction Tendency Scale and Bedtime Procrastination Scale were used to measure sleep and psychological condition of 1,048 college students.

**Results:**

Family cohesion negatively predicted bedtime procrastination. Additionally, positive coping style and mobile phone addiction had significant independent mediating effects. Furthermore, positive coping style and mobile phone addiction had chain mediating effects between family cohesion and bedtime procrastination.

**Conclusion:**

This study revealed the effect of coping styles and mobile phone addiction on the relationship between family cohesion and bedtime procrastination among Chinese college students. These findings explained the mechanisms of bedtime procrastination from the perspective of environment, so as to effectively intervene the bedtime procrastination of college students from the perspective of external environment.

## Introduction

Recently, insufficient sleep has become an extremely serious social problem. Severe insufficient sleep can lead to depression, anxiety, endocrine system and metabolic disorders, cognitive impairment, and increased risk of self-harm and suicide [1–4]. According to *‘Annual Sleep Report of China 2022’*, it was found that individuals habituated delaying their bedtime has become the main cause of insufficient sleep, and 54.3% of college students reported that they had a certain degree of bedtime procrastination [5]. Bedtime procrastination, as a special form of procrastination, refers to the behavior of an individual who is unable to go to bed at the scheduled time without any external factors [6]. Research has found that bedtime procrastination is likely to lead to sleep-related problems such as poor sleep quality, short sleep duration, and insomina [6, 7]. In addition to its impact on individual sleep status, bedtime procrastination will also have a significant impact on anxiety, depression, daytime function and subjective well-being [8, 9]. More seriously, sleep procrastination is also a risk factor for sleep disorders [10], which has extremely serious harm to an individual’s sleep and mental health. Adequate sleep is the basis for college students to have a healthy body and mind. How to make college students go to bed at the scheduled bedtime to ensure their physical and mental health is a problem worthy of in-depth discussion. Therefore, the present study intends to explore the occurrence mechanisms of bedtime procrastination in college students, in order to provide research support for the prevention and intervention of bedtime procrastination for college students.

Most of the research on bedtime procrastination has focused on the internal psychological mechanisms, but neglected to explore the environmental factors that contribute to healthy sleep. According to the theory of the opposing process of sleep, sleep is a physiological state in which the awareness and responsiveness to the external environment are fundamentally weakened [11]. Therefore, sleep behavior should be performed in a safe environment or without vigilance. Family cohesion refers to the extent to which individuals feel emotionally bonded to family members [12]. Research found that high intimacy individual would perceive higher levels of security and play a protective role in sleep disorders caused by insecurity [13], the fact of that is high intimacy can create a safe and alert-free environment to promote sleep. Moreover, improving family cohesion is an effective intervention to alleviate sleep problems among college students during the COVID-19 pandemic [14]. Compared with families with high family cohesion, dysfunctional families are unable to adequately promote the correct development of children’s sleep habits due to their disordered state [15, 16], and their parents often fail to act as role models, leading to poor awareness of sleep hygiene in children and adolescents [17]. The family environment of childhood also affects an individual’s sleep and academic performance in college [18]. Lack of sleep health knowledge may lead to irregular sleep habits and sleep problems [19, 20]. Therefore, it can be inferred that individuals with high family cohesion have better sleep hygiene habits and night sleep status, thus reducing the possibility of bedtime procrastination. Hypothesis: family cohesion negatively predicts bedtime procrastination (H1).

Coping style refers to the means used by individuals to adapt to situations and maintain emotional stability when faced with environmental requirements and emotional disturbances, which can be divided into positive coping styles and negative coping styles [21]. Researches on the relationship between family cohesion and coping styles found that individuals in environments with high family cohesion tended to adopt a more mature and stable way to deal with events [22], while individuals in the absence of family cohesion tended to adopt a negative coping style [23]. There is a strong correlation between coping styles and emotional well-being. Cai et al. [24] found that positive coping styles can help individuals stabilize their emotions and generate positive emotion, while negative coping styles are related to negative emotion such as depression and anxiety, which can promote the individual’s bedtime procrastination behavior [25].Therefore, we speculated that a high level of family cohesion would help individuals stabilize their emotions by coping with stressful events in a good way, thus reducing their bedtime procrastination behavior. Hypothesis: Coping styles mediate the relationship between family cohesion and bedtime procrastination (H2).

According to the compensatory Internet use theory, individuals’ use of the Internet or mobile phone to fill the inner void will be influenced by the intimate relationship in life, individuals with low family intimacy will fill their inner void through frequent mobile phone use [26]. Empirical research also have shown that family cohesion is negatively correlated with mobile phone addiction in college students, which is mainly reflected in the withdrawal and loss of control of mobile phone addiction [27].The survey on the use of mobile phones before sleeping among Chinese college students found that 96.8% of them had the habit of using mobile phones before sleep, and more than 70% of them still used mobile phones late at night [28]. Individuals using mobile phones for online social networking and entertainment had become one of the main reasons for bedtime procrastination among Chinese college students [29]. The Internet gratification theory [30] suggested that individuals could obtain satisfaction and happiness through the use of mobile phones. However, as the frequency of mobile phone use increases, the satisfaction decreases. In order to obtain a balance of the satisfaction and happiness with the previous situation, individuals will continuously increase the amount of time using mobile phones. Therefore, individuals generally tend to delay going to bed in order to have more time to use mobile phones to satisfy themselves [31, 32]. A recent longitudinal study also confirmed that front-side phone addiction significantly predicted post-test bedtime procrastination [33]. Accordingly, the present study speculated that mobile phone addiction may play a mediating role in family cohesion and bedtime procrastination (H3).

Family cohesion is very important to each family member, and good family cohesion helps create a good family environment and conducive to communication between family members [34]. Therefore, a high level of family cohesion is beneficial to develop positive coping styles and prepare for upcoming stressful events with a positive and open attitude [35]. Coping styles play an important role in addictive behaviors. According to the stress assessment coping theory [36], avoidance behaviors that individuals engage in to avoid threats can induce addictive behaviors because addictive behaviors can provide temporary relief either cognitively or behaviorally. Consistent with this, research has also found that negative avoidance coping styles positively predict mobile phone addiction, while positive coping styles negatively predict mobile phone addiction [37]. In addition, excessive use of mobile phones at night will lead to abnormal secretion of melatonin due to the blue light released by mobile phone screens, and then appear the phenomenon of increased sleep latency and delayed sleep onset time [31, 38]. Therefore, this study speculated that coping style and mobile phone addiction played a chain-mediating role in the relationship between family cohesion and bedtime procrastination (H4).

## Materials and methods

### Participants

There were 1048 college students participated in this study, including 585 male (55.8%) and 463 female (44.2%). The mean age of the participants was 20.25 years a standard deviation of 2.29 years, including 361 (34.4%) freshmen, 207 (19.8%) sophomores, 118 (11.3%) juniors, 183 (17.5%) seniors, and 179 (17.1%) graduate students and above. There were 520 (49.6%) only children and 528 (50.4%) not only children. None of the participants had sleep disorders or psychosomatic disorders.

### Measures

#### Family cohesion scale (FCS)

Family Cohesion Scale modified by Fei et al. [39] was used to measure family cohesion. The scale contains 16 items, all of which are scored from 1 to 5 points, in which 1 means “never” and 5 means “always”. The higher the score, the higher the level of family cohesion. In this study, the Cronbach’s α coefficient of this scale was 0.885.

#### Simplified Coping Styles Questionnaire (SCSQ)

Simplified Coping Styles Questionnaire modified by Xie [40] was used to measure the coping styles of the participants. The scale is divided into two dimensions, positive coping style and negative coping style, and contains a total of 20 items. The scale is scored by four points, in which 1 means “do not take” and 4 means “often take”. The higher the score on positive coping style, the higher the tendency of the individual to adopt positive coping style. In this study, the Cronbach’s α coefficient of this scale was 0.895, among which, the Cronbach’s α coefficient of positive subscale was 0.902, and the Cronbach’s α coefficient of negative subscale was 0.859. The Cronbach’s α coefficient of the packaged positive subscale was 0.891, and the Cronbach’s α coefficient of the packed negative subscale was 0.878.

#### Mobile Phone Addiction Tendency Scale (MPATS)

The level of the mobile phone addiction was measured by the Mobile Phone Addiction Tendency Scale revised by Xiong et al. [41]. The scale contains a total of 16 items, including four dimensions of withdrawal symptoms, prominent behavior, social comfort and mood change. Likert 5-point score is adopted, where 1 represents “very consistent” and 5 represents “very inconsistent”. The higher the total score, the higher the individual’s level of phone addiction. The Cronbach’s α coefficient of this scale in this study was 0.934. The Cronbach’s α coefficient of withdrawal symptoms, salience, social comfort and mood changes subscales were 0.842, 0.844, 0.847 and 0.712, respectively.

#### Bedtime Procrastination Scale (BPS)

Bedtime Procrastination Scale revised by Ma et al. [42] was adopted to measure the severity of individual bedtime procrastination. The scale is a single-dimensional structure with nine items, and Likert 5 points were used to score, with 1 representing “completely inconsistent” and 5 representing “completely consistent”. The higher the score, the more severe the individual’s bedtime procrastination. Cronbach’s α value of this scale in this study was 0.846.

### Statistical analyses

SPSS 22.0 was used for common method bias test, descriptive statistical analysis and Person correlation analysis. Amos 24.0 was used to construct a structural equation model to test the multiple mediating effects between family cohesion and bedtime procrastination. The percentile Bootstrap method with deviation correction was used to test the significance of the mediation effect. If the 95% confidence interval of Bootstrap did not contain 0, the effect was significant.

## Result

### Harman single factor test and descriptive statistics

Harman single factor test was used to test the common method bias [43]. The results showed that there were nine factors with characteristic roots greater than 1, and the variance explained by the first factor was 18.88%, which was less than the critical value of 40%, indicating that there was no serious common method bias in this study.

Descriptive statistics of family cohesion, coping styles, mobile phone addiction, and bedtime procrastination are shown in Table 1. The results showed that family cohesion was positively correlated with coping style, and negatively correlated with mobile phone addiction and bedtime procrastination. Coping style was positively correlated with mobile phone addiction, but weakly negatively correlated with bedtime procrastination. Mobile phone addiction was positively associated with bedtime procrastination.

**Table 1:** Descriptive statistics of each variable and correlation coefficient matrix

	M ± SD	1	2	3	4	5	6	7	8	9	10
1 Family cohesion	34.15 ± 10.62	1									
2 Coping style	2.82 ± 0.51	0.31^***^	1								
3 Mobile phone addiction	2.61 ± 0.88	-0.15^***^	0.15^***^	1							
4 Bedtime procrastination	3.12 ± 0.82	-0.26^***^	-0.07^*^	0.38^***^	1						
5 Abstinence syndrome	4.25 ± 1.39	-0.09^**^	0.14^***^	0.94^***^	0.35^***^	1					
6 Conspicuous behavior	2.32 ± 1.00	-0.15^***^	0.13^***^	0.90^***^	0.35^***^	0.79^***^	1				
7 Social comfort	2.03 ± 0.81	-0.19^***^	0.06^*^	0.81^***^	0.32^***^	0.68^***^	0.64^***^	1			
8 Mood change	1.84 ± 0.75	-0.11^***^	0.17^***^	0.87^***^	0.31^***^	0.75^***^	0.75^***^	0.61^***^	1		
9 Positive coping style	3.07 ± 0.55	0.42^***^	0.84^***^	-0.06^*^	-0.20^***^	-0.04	-0.06^*^	-0.12^***^	-0.02	1	
10 Negative coping style	2.43 ± 0.72	0.06	0.79^***^	0.33^***^	0.10^**^	0.30^***^	0.31^***^	0.25^***^	0.32^***^	0.33^***^	1

Note: * *p* < 0.05 ** *p* < 0.01 *** *p* < 0.001

### Structural equation model of the relationship between variables

**Fig. 1:**
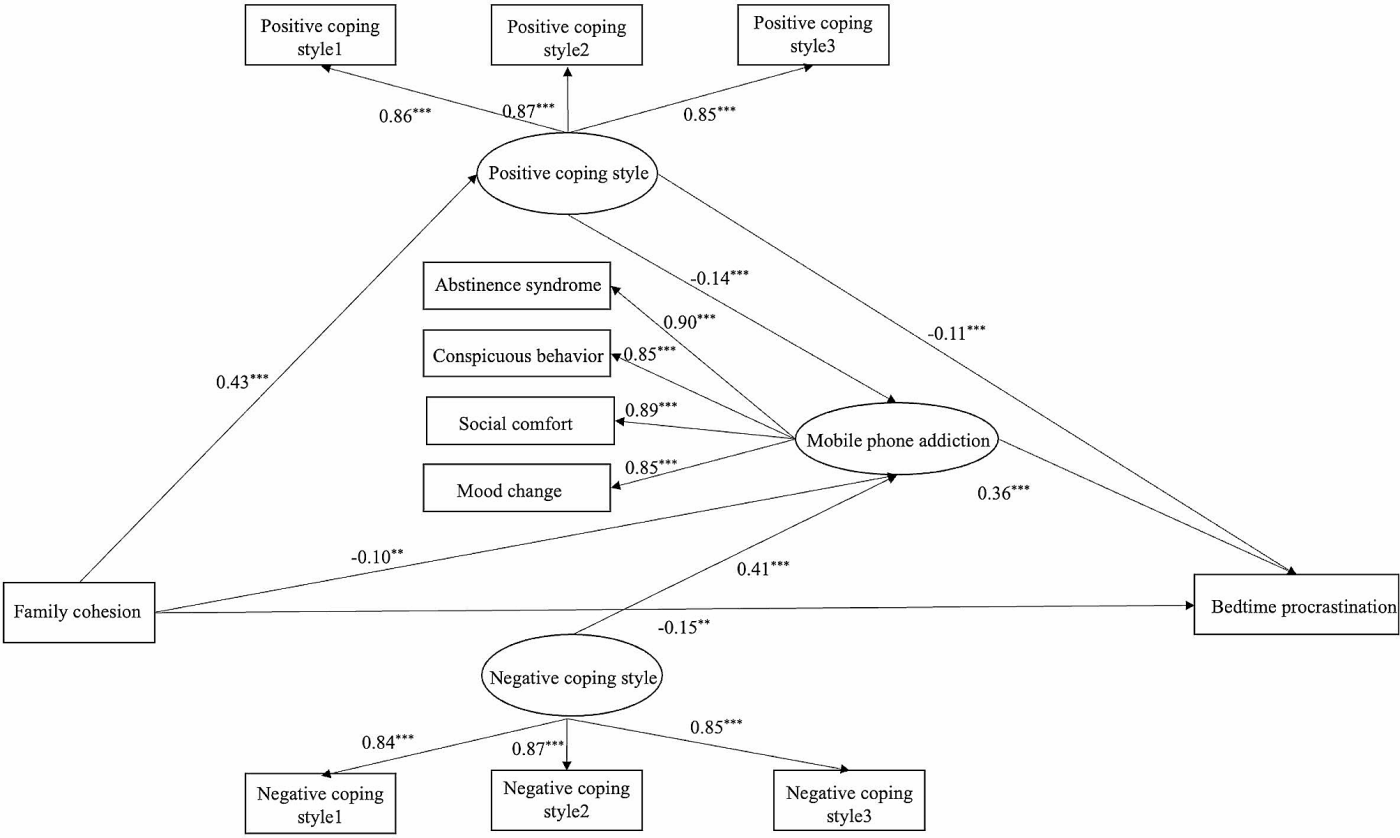
The chain mediating effect of family closeness on bedtime procrastination

In order to avoid the expansion of measurement error, the balance orientation method is adopted to package the positive coping style and the negative coping style. There are four dimensions of mobile phone addiction, which are packaged according to the dimensions. Initial model M1 was constructed according to the hypothesis, with family cohesion as the independent variable, bedtime procrastination as the dependent variable, and positive coping style, negative coping style and mobile phone addiction as the mediating variables. AMOS24.0 was used to test the model, and the initial model had a good fit (Table 2), but it was found that the direct path of family cohesion on negative coping style (*β* = 0.01, *P* > 0.05) and negative coping style on bedtime procrastination (*β* = 0.11, *P* > 0.05) was not significant. Therefore, the initial model M1 was modified, and the competition model was first established. The path from negative coping style to bedtime procrastination was set as 0, and the competition model M2 was established. All indexes of the model were well fitted. Then, the path from family cohesion to negative coping style was set as 0, and the competition model M3 was established. According to the nested model comparison theory [44], the competition model M3 is compared to the initial model M1,Δ*Χ*^2^ (2) = 1.71 *P* > 0.05, indicating that the Chi-square value of the competition model has not been significantly improved. Based on the principle of model minimalism, the competing model M3 is considered to be the better model (Fig. 1).

**Table 2:** Structural equation model fitting index

Model	*Χ* ^2^	df	*Χ*^2^/df	GFI	NFI	IFI	TLI	CFI	RMSEA
M1	240.35	47	5.11	0.96	0.97	0.97	0.96	0.97	0.06
M2	240.77	48	5.02	0.96	0.97	0.97	0.96	0.97	0.06
M3	242.06	49	4.94	0.96	0.97	0.97	0.96	0.97	0.06

### Mediation analysis

Based on the structural equation model shown in Fig. 1, the non-parametric percentile Bootstrap method with deviation correction was used for 5000 repeated samples to test the mediation effect. If the 95% confidence difference of the mean path coefficient shown in Fig. 1 did not include 0, it indicated that the mediation effect was significant. Combined with Fig. 1; Table 1, it can be seen that family cohesion exerts an indirect mediating effect on bedtime procrastination through positive coping style and mobile phone addiction. Positive coping style has a significant mediating effect between family cohesion and bedtime procrastination; Mobile phone addiction has a significant mediating effect between family cohesion and bedtime procrastination; Family cohesion predicted bedtime procrastination through the chain mediating effect of positive coping style and mobile phone addiction. This study has found a multi-mediator model of family cohesion on bedtime procrastination [45]. By comparing the path coefficients, we found that among the three mediating paths (Table 3), the path effect mediated by positive coping style was the strongest (*β* = −0.05, *P* < 0.01). However, the pathway effect mediated by mobile phone addiction (*β* = −0.04, *P* < 0.01) and the pathway effect mediated by positive coping style and mobile phone addiction (*β* = −0.02, *P* < 0.001) were weak.

**Table 3:** Bootstrap analysis of mediating effect significance

Pathway	Standardization Indirect Effect	Proportion of Indirect Effect	95% confidence interval		*P*
			LLCI	ULCI	
Family cohesion→Positive coping style→Bedtime procrastination	(0.43) × (− 0.11) = -0.05	15.63%	−0.08	−0.02	0.00
Family cohesion→Mobile phone addiction→Bedtime procrastination	(− 0.10) × (0.36) = − 0.04	12.50%	−0.06	−0.01	0.00
Family cohesion→Positive coping style→Mobile phone addiction→Bedtime procrastination	(0.43) × (− 0.14) × (0.36) = − 0.02	6.25%	−0.04	−0.01	0.00
Total Indirect effect	−0.11	34.38%	−0.14	−0.07	0.00

## Discussion

The present study examined the relationship between family cohesion, coping styles, mobile phone addiction and bedtime procrastination among Chinese college students and further explored the specific mechanism by which family cohesion influences bedtime procrastination. Our results indicated that coping styles and mobile phone addiction had respective and serial mediation effects in the association between family cohesion and bedtime procrastination, which supports our hypotheses. These findings underscore the importance of coping styles and mobile phone additcion as potential factors in explaining the relationship between family cohesion and bedtime procrastination among Chinese college students. This study is conducive to a deeper understanding of the mechanisms underlying bedtime procrastination in college students and provides a scientific basis for relieving the issue of bedtime procrastination and intervening in its occurrence.

The present study is the first to investigate the mechanisms of bedtime procrastination from the perspective of family environment. The results show that family cohesion significantly negatively predicts bedtime procrastination. In the context of high pressure, individuals with a high level of family cohesion have a more harmonious and warm family environment and a higher level of parental support, which is conducive to creating a safe atmosphere and experiencing a higher level of security [46]. On the contrary, in families lacking family cohesion, individuals feel more depressed, lonely and insecure [47–49], which is closely related to hypervigilance and frequent night awakenings [50]. Moreover, long-term exposure to a poor family environment disrupts the sleep-wake pattern of adolescents and leads to various sleep problems [51]. Therefore, a possible explanation for the relationship between the family cohesion and bedtime procrastination is that individuals with high family cohesion have better sleep hygiene habits and night sleep status [15–17, 19, 20], which allows individuals to go to bed on time and prevent bedtime procrastination.At the same time, the results of this study suggest that in the prevention and intervention of bedtime procrastination, we should not only pay attention to the internal factors of individuals, but also pay attention to the external environmental factors, especially the influence of family environment on bedtime procrastination. In the future, intervention techniques for improving families can also be applied to improve and prevent bedtime procrastination to a certain extent.

Second, we found that family cohesion positively predicted positive coping styles, which was consistent with previous research [34]. Moreover, the present study further found that positive coping style was an independent mediator between family cohesion and bedtime procrastination. In families with high family cohesion, there is usually a more stable family structure, supportive parent-child relationship and warm family atmosphere [52], which will enable college students to cope with environmental requirements and emotional troubles in a more positive way. In addition, college students with positive coping style will use adaptive emotion regulation strategies to cope with emotional disturbance before going to bed, which is conducive to the stability of individual emotions and the generation of positive emotions, and thus effectively prevent the occurrence of bedtime procrastination and avoid using bedtime procrastination as a means of emotion repair [25]. Different from the hypothesis and previous studies, our results didn’t find an independent mediating effect or chain mediating effect of negative coping styles on the relationship between family cohesion and bedtime procrastination.

Furthermore, we found an independent mediating effect of mobile phone addiction between family cohesion and bedtime procrastination. Specifically, individuals with low family cohesion were more likely to be addicted to mobile phones, which leaded to individuals more likely to have bedtime procrastination behaviors. In families lacking family cohesion, college students seek to fill the inner void through Internet or mobile phone use due to their own loneliness and negative emotional needs [53, 54], which is consistent with the compensator Internet use theory. However, the Internet gratification theory also points out that although individuals can obtain satisfaction and happiness by using mobile phones, their satisfaction will decrease with the increase of the frequency of using mobile phones. In order to get a balanced satisfaction and happiness again, individuals will keep increasing their time of using mobile phones. In general, individuals tend to increase their use of mobile phones before bedtime, which contributes to bedtime procrastination [31].

Finally, this study further found the chain mediating effect of positive coping style and mobile phone addiction on family cohesion and bedtime procrastination. More specifically, students with higher family cohesion more likely to adopt more positive coping styles, which result in less mobile phone addiction and ultimately less bedtime procrastination. Therefore, family cohesion can not only avoid the phenomenon of increased sleep latency caused by the influence of blue light to a certain extent, but also help reduce the appearance of bedtime procrastination. This study provides a new approach for the intervention of bedtime procrastination from the perspective of family environment. Family cohesion, as a protective factor for bedtime procrastination, emphasizes the degree of emotional cohesion among family members. Close family cohesion can help individuals establish positive coping styles, so as to avoid mobile phone addiction and bedtime procrastination.

In summary, our study had several strengths including the first to shed light on the association between family cohesion and bedtime procrastination among Chinese college students and novel findings of respective and serial mediation effects of coping styles and mobile phone addiction in the association between family cohesion and bedtime procrastination. However, this study still has some shortcomings: First of all, it is a cross-sectional study, and the causal relationship between variables cannot be inferred. In the future, experimental or longitudinal study is still needed to explore the mechanism of family cohesion on bedtime procrastination. Secondly, the participants of this study are college students, whose education level has a great influence. The increase of their knowledge and experience may reduce the influence of their natural family on them, which has certain particularity in exploring the relationship between family cohesion and bedtime procrastination, which limits the promotion of the research results to a certain extent. The results of this study need to be validated for different groups in the future. Third, the convenient sampling method adopted in this study will reduce the accuracy of sampling and therefore poor representativeness. Therefore, in the future, sample groups and sampling areas should be expanded on this basis, and the research results should be further verified and investigated in different groups and regions. Fourth, we did not control for other sleep-related variables, such as sleep duration and sleep quality. Future research should further explore the applicability of this model on the basis of strict control of irrelevant variables. Last but not least, the effect of family cohesion on bedtime procrastination may also be accounted for by other factors, such as negative emotions and chronotype. Future research should consider examining the mediating effects of factors other than coping styles and mobile phone addiction on the relationship between family cohesion and bedtime procrastination.

## Data Availability

The data that support the findings of this study are available on request from the corresponding author.
